# Artificial intelligence-assisted echocardiographic monitoring in pediatric patients on extracorporeal membrane oxygenation

**DOI:** 10.3389/fcvm.2024.1418741

**Published:** 2024-12-11

**Authors:** Weiling Chen, Jinhui Wu, Zhenxuan Zhang, Zhifan Gao, Xunyi Chen, Yu Zhang, Zhou Lin, Zijian Tang, Wei Yu, Shumin Fan, Heye Zhang, Bei Xia

**Affiliations:** ^1^ Department of Ultrasonography, Shenzhen Children’s Hospital, Shenzhen, China; ^2^ School of Biomedical Engineering, Sun Yat-sen University, Shenzhen, China

**Keywords:** artificial intelligence, echocardiography, critical monitoring, ECMO, pediatrics, left ventricular function

## Abstract

**Background:**

Percutaneous extracorporeal membrane oxygenation (ECMO) is administered to pediatric patients with cardiogenic shock or cardiac arrest. The traditional method uses focal echocardiography to complete the left ventricular measurement. However, echocardiographic determination of the ejection fraction (EF) by manual tracing of the endocardial borders is time consuming and operator dependent. The standard visual assessment is also an inherently subjective procedure. Artificial intelligence (AI) based machine learning-enabled image analysis might provide rapid, reproducible measurements of left ventricular volumes and EF for ECMO patients.

**Objectives:**

This study aims to evaluate the applicability of AI for monitoring cardiac function based on Echocardiography in patients with ECMO.

**Materials and methods:**

We conducted a retrospective study involving 29 hospitalized patients who received ECMO support between January 2017 and December 2021. Echocardiogram was performed for patients with ECMO, including at pre-ECMO, during cannulation, during ECMO support, during the ECMO wean, and a follow up within 3 months after weaning. EF assessment of all patients was independently evaluated by junior physicians (junior-EF) and experts (expert-EF) using Simpson's biplane method of manual tracing. Additionally, raw data images of apical 2-chamber and 4-chamber views were utilized for EF assessment via a Pediatric ECMO Quantification machine learning-enabled AI (automated-EF).

**Results:**

There was no statistically significant difference between the automated-EF and expert-EF for all groups (*P* > 0.05). However, the differences between junior-EF and automated-EF and expert-EF were statistically significant (*P* < 0.05). Inter-group correlation coefficients (ICC) indicated higher agreement between automated-EF and expert manual tracking (ICC: 0.983, 95% CI: 0.977∼0.987) compared to junior assessments (ICC: 0.932, 95% CI: 0.913∼0.946). Bland–Altman analysis showed good agreements among the automated-EF and the expert-EF and junior-EF assessments. There was no significant intra-observer variability for experts' manual tracking or automated measurements.

**Conclusions:**

Automated EF measurements are feasible for pediatric ECMO echocardiography. AI-automated analysis of echocardiography for quantifying left ventricular function in critically ill children has good consistency and reproducibility with that of clinical experts. The automated echocardiographic EF method is reliable for the quantitative evaluation of different heart rates. It can fully support the course of ECMO treatment, and it can help improve the accuracy of quantitative evaluation.

## 1 Introduction

Extracorporeal membrane oxygenation (ECMO) is crucial for treating children with severe cardiopulmonary disease. It allows temporary support for pulmonary and/or cardiac failure refractory to conventional therapy (1, 2). ECMO involves draining blood from the body, oxygenating it in a machine, and then returning it to the body. This process alters cardiac volume loading and lung perfusion. Echocardiography plays an important role in ECMO. It provides essential diagnostic and anatomical information before ECMO initiation. Echocardiography guides the selection of the ECMO mode (Veno-Arterial for cardiorespiratory support; Veno-Venous for respiratory support alone) and aids in safe and efficient ECMO cannula positioning. It can also assist in flow optimization and decompression of left-side structures during the ECMO run. It offers a modality for rapid troubleshooting and patient evaluation and facilitates decision-making for eventual weaning from ECMO support (3, 4). However, prolonged ECMO cardiopulmonary bypass increases the risk of complications such as infection, bleeding, and thrombosis. Therefore, high-frequency echocardiography is necessary to monitor changes in a patient's condition continuously.

Echocardiography has shifted from monitoring cardiac function to becoming a clinical tool integrated into treatment evaluation and management. It is used to assess indications in ECMO and select the appropriate support mode. Echocardiography has also emerged as a risk stratification tool for evaluating ventricular function and predicting the degree of potential ventricular recovery (5). Due to its non-ionizing radiation nature and bedside accessibility, echocardiography is extensively utilized for noninvasive monitoring of cardiac function in critically ill children. However, echocardiographic monitoring at the point of care demands swift and frequent comparisons. It is susceptible to variations in imaging quality and operator subjectivity. This may exhibit significant inter- and intra-observer variability without sufficient training and experience (6, 7).

Artificial intelligence (AI) has been employed to automatically calculate the pertinent parameters of the left ventricle (LV) in echocardiography. Initially, research primarily concentrated on automatically segmenting the LV in echocardiography, employing deep learning semantic algorithms (8). Researchers have suggested utilizing datasets like CAMUS and Echo Net-Dynamic to advance deep learning for a broader array of clinical applications. These datasets enable researchers to pretrain deep learning models and enhance model robustness (9, 10). Additionally, researchers have delved into the potential of deep learning algorithms for analyzing echocardiography data. They utilize deep learning models for automated analysis of point-of-care ultrasound and multi-disease datasets (11, 12). Nevertheless, the majority of these studies have focused on adult echocardiography, with limited investigation into pediatric echocardiography. Pediatric echocardiography encompasses subjects spanning from neonates to young adults, with weights ranging from 3 to 70 kg, heart rates varying from 60 to 150 bpm, and cardiac masses between 25 and 350 g. The significant variability in children's heart sizes and heart rates, combined with their poor coordination, amplifies the challenge of quantitative analysis in echocardiography. Discrepancies arise when the frequency of a fan-sweep probe fails to align with the increase in an infant's heart rate, leading to substantial deviations in the visual assessment of the heart's structure and function, consequently yielding inaccurate calculations.

We previously developed an AI algorithm capable of automatically processing and extracting imaging features in echocardiography (13). We utilized this model for the automatic detection of LV systolic function in pediatric patients on ECMO to ascertain their cardiac function levels. Subsequently, we compared the results with those obtained by experienced echocardiographers to assess whether AI assistance could enhance accuracy.

## 2 Materials and methods

### 2.1 Pediatric ECMO quantification network

**Figure 1:**
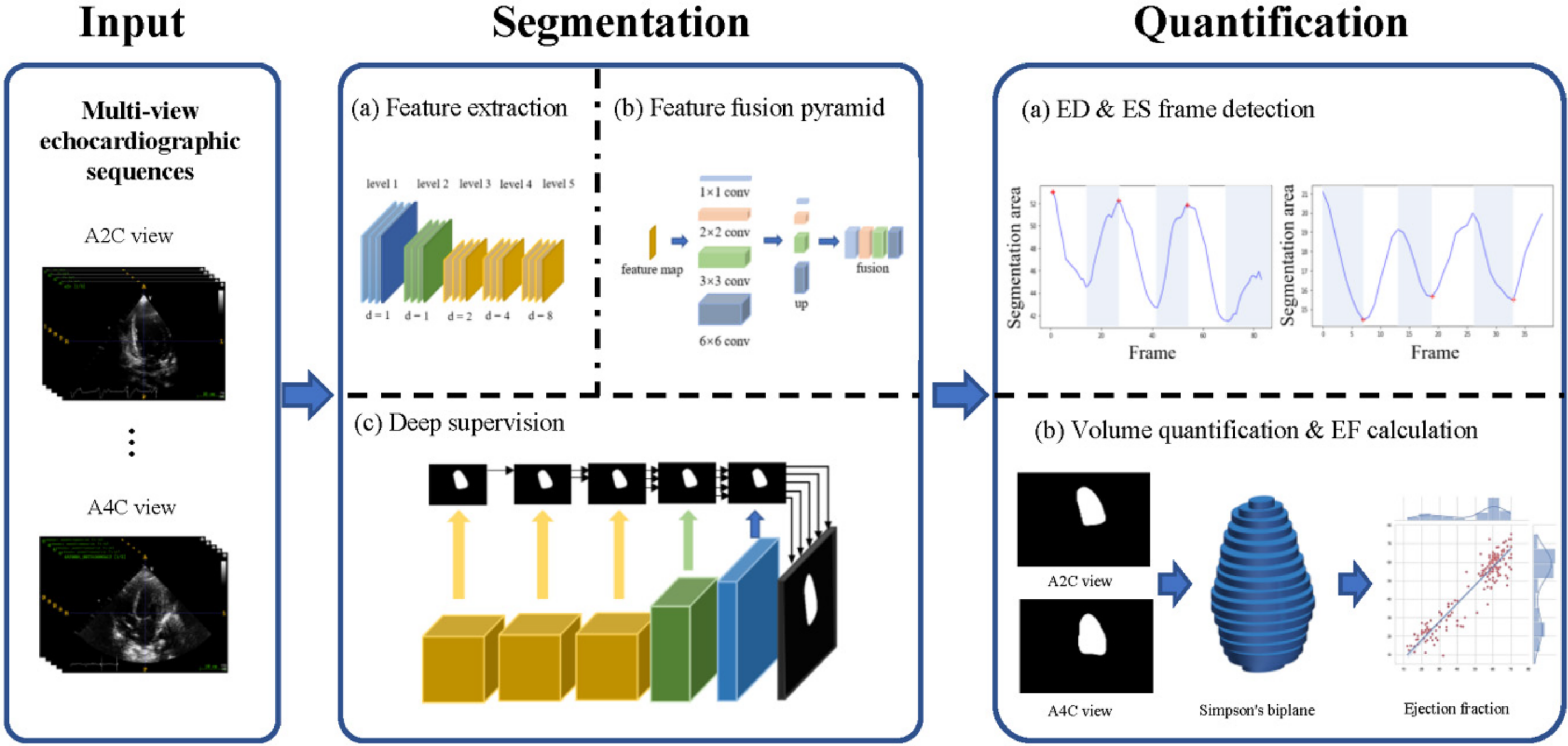
Overview of the PEQ-Net workflow. In the input part, we use two echocardiographic views, A2C and A4C. The two views echocardiographic sequences are segmented and quantified at the same time by a unified model. The segmentation part is divided into three stages: **(a)** Feature extraction, **(b)** Feature fusion pyramid, **(c)** Deep supervision. This part outputs a frame-by-frame segmentation of the echocardiographic sequence. In the quantification part, ED frames and ES frames are obtained based on the detection of the peak area of each frame in the sequence. Then, we use Simpson's biplane method for volume quantification. Further ED and ES volumes and ejection fractions can be obtained for each case.

The study used the Pediatric ECMO Quantification model (PEQ-Net) based on DPS-Net (12), as illustrated in Figure 1. PEQ-Net comprises three main components in the architecture for the task of LV segmentation in this study. First, the feature extraction module employs a multi-scale and multi-level network comprising five levels of encoders to extract both low-level detail information and high-level semantic information. Within the low-level encoders, features are extracted from fine regions of the image, preserving image details and refining image mask boundaries. Conversely, the high-level encoders focus on extracting high-level semantic information and capturing global features of the image. Within each encoder, a dense concatenation design is employed to merge the feature map of the previous level with the feature map of the current level, thereby integrating low-level information with high-level information. In high-level feature extraction, dilated convolution is utilized to replace original pooling operations and reduce image size. Each level employs a different dilation rate (1, 1, 2, 4, and 8). This dilated convolution design enables the network to expand its receptive field without sacrificing image details due to size reduction.

Second, the multi-scale and multi-level feature fusion module combines the high-level and low-level features. A feature pyramid network facilitates the connection between the encoder and the decoder. It employs four parallel convolutions and encompasses four distinct scales of perceptual fields to produce feature maps. These feature maps are then up-sampled to the same resolution and concatenated into a block feature map. This module effectively captures both the global structure and small local variations of the LV.

Third, the feature maps are supervised at each level in deep supervision. This stage decodes the feature maps from the output of each feature extraction module. The multi-scale feature maps are up-sampled to a uniformly sized feature map with different up-sampling rates (16, 8, 4, 2, 1), resulting in five activated feature maps. These activated feature maps are then compared with the ground truth to compute five loss values. All five loss values utilize the same composite loss function, which comprises three individual loss functions: dice loss, cross-entropy loss, and mean absolute error loss. The equation for the composite loss function is as follows:

(1) $$\begin{array}{c} L_{COMP}=L_{WCE}+L_{GDICE}+L_{MMAE} \end{array}$$

$L_{WCE}$ is the weighted binary cross-entropy loss, $L_{GDICE}$ is the generalized dice loss, and $L_{MMAE}$ is the modified mean absolute error loss. The definitions of the loss functions are:

(2) $$\begin{array}{c} L_{WCE}(PRED,GT)=-w\cdot GT\cdot \log(PRED)-(1-GT)\cdot \log(1-PRED) \end{array}$$

(3) $$\begin{array}{c} L_{GDICE}(PRED,GT)=1-2\cdot \frac{a\cdot GT\cdot PRED+(1-GT)\cdot (1-PRED)}{a\cdot (GT+PRED)+2-GT-PRED} \end{array}$$

(4) $$\begin{array}{c} L_{MMAE}(PRED,GT)=\log(1+e^{\left|PRED-GT\right|}) \end{array}$$

Where PRED is prediction, GT is ground truth, *w* and *a* are balance weights. These three loss functions are utilized to optimize the pixel-level similarity, overlapping degree, and spatial Euclidean distance between the predicted segmentation results and the ground truth, respectively.

All five losses are propagated back to optimize the model parameters. This allows the extraction and fusion of high- and low-level features to be supervised simultaneously, and it reduces the risk of over fitting.

The quantitative assessment of the LV can be divided into two main parts: end-diastolic (ED) and end-systolic (ES) frame detection and volume quantification. The ED and ES frame detection algorithm calculates the predicted LV segmentation area in two views (A2C and A4C) and then obtains the ED and ES frames by comparing the maximum and minimum frames in these two views. We used the traditional Simpson biplane disc method for LV volume calculation.

All images were up-sampled to their original size. We need to unify the size of the original images to satisfy the requirement of training the PEQ-Net. The unified size is (256 × 256). We need to calculate the quantitative metrics between the predicted images and the original ground truth images. The original size is (636 × 434). The original size of the images is bigger than the unified size. To evaluate the performance of models precisely, the predicted images should be consistent with the ground truth images. Therefore, we up-sample the images with the unified size to their original size.

A stochastic gradient descent method with a momentum of 0.9 was applied to minimize the combined error of the LV segmentation. The initial learning rate was 0.002, decreasing by a factor of 0.5 every 50 epochs. The study adopted the Pytorch programming framework (14) to build the model. All calculations were performed on a workstation with a Xeon 2.90 GHz CPU and an NVIDIA RTX A6000 GPU under the Ubuntu 18.04 operating system.

### 2.2 Model training and validation

The DPS-Net, a convolutional neural network (CNN), was constructed upon a modified U-net architecture with a symmetric design. It aimed to achieve rapid and accurate semantic segmentation. The development of DPS-Net utilized 36,890 frames of two-dimensional echocardiography from 340 patients, subsequently undergoing testing on both the CAMUS dataset and the Echo Net-Dynamic dataset (9, 10). The datasets are openly accessible. The DPS-Net exhibited high performance on a large-scale dataset, indicating its significant adaptability across various echocardiographic systems.

Our dataset consisted of 11,718 frames of two-dimensional echocardiography obtained from 80 healthy children. It was utilized for algorithm development. Echocardiography procedures were conducted using VIVID E9 and E95 machines from General Electric, Milwaukee, WI. All datasets were anonymized and saved in DICOM format. Frame-by-frame labeling of the dataset was performed by a team of 8 heart specialists. The labels underwent further review by three senior experts. We used the DPS-Net as a pretrained model due to differences between adult and pediatric data. Then, we fine-tuned the weights using the pediatric dataset, resulting in a model named PEQ-Net.

The study adopts stratified sampling to split data according to patient number. The study splits 60% of the total patient number as the training dataset randomly. The study splits 20% of the total patient number as the validation dataset randomly. The remaining 20% of the total dataset is the test dataset.

### 2.3 Patients and imaging protocol

The study included 29 pediatric patients on ECMO from the intensive care unit of Shenzhen Children's Hospital from January 2017 to January 2022. Ethical approval was obtained for the study [Ethics Approval 2020012].

All echocardiographic scans were conducted using a GE Vivid echocardiography system (GE Healthcare, Milwaukee, WI). The system was equipped with M5S and M6S probes, featuring frequency ranges of 1.4–4.6 MHz and 2.5–6.4 MHz, respectively. The data were digitally recorded to facilitate offline measurements. The monitoring protocol followed ECMO echocardiography guidelines. Bedside dynamic echocardiographic monitoring was conducted before intubation, during support, before weaning, and after weaning. Apical four-chamber views (A4C) and apical two-chamber views (A2C) were acquired at the bedside, measured in real-time, and digitally stored. The images routinely encompassed 1 to 3 cardiac cycles, with a dataset comprising 184 instances featuring three cycles and 2 instances with a single cycle, and provided complete left ventricular end-diastolic volume (LVEDV), end-systolic volume (LVESV), and ejection fraction (EF) results (referred to as junior-EF). Both AI models and echocardiography experts utilized these images for offline quantitative analysis. The AI algorithm automatically estimated EF (automated-EF) from the A4C and A2C view echocardiography. While echocardiography experts performed offline measurements to calculate EF (expert-EF).

In total, 218 echocardiograms were conducted across all patients. Each of these 218 echocardiographic examinations was evaluated for junior-, automated-, and expert-EF, respectively. Junior-EF measurements were attainable for all patients. However, expert-EF measurements were not feasible in 27 datasets, resulting in a feasibility rate of 87.6%. Similarly, fully automated-EF measurements were not feasible in 32 datasets, yielding a feasibility rate of 85.3%. The infeasibility is attributed to several factors, including non-standard image acquisition, poor delineation of the endocardial border, incomplete image sets, and the presence of arrhythmias such as ventricular fibrillation and frequent premature beats. Consequently, the study encompassed 186 datasets that were ultimately included in the final analysis.

### 2.4 Automatic image analysis

After inputting the A4C and A2C view echocardiography to the PEQ-Net, the model automatically captured the ED and ES. And it automatically calculated the LVEDV, LVESV, and automated-EF. We compared LV segmentations between manual and automatic methods to evaluate the accuracy of automatic LV segmentation. Metrics such as the Jaccard, Dice coefficient, precision, recall, and Hausdorff distance (HD) were utilized. Jaccard and Dice coefficients quantify the similarity between the ground truth and the segmentation outcomes by measuring their intersection over their union. Precision and recall metrics evaluate the correctness and exhaustiveness of the positive pixel predictions made by the segmentation algorithm. Furthermore, the HD assesses the maximum boundary discrepancy between the segmentation results and the ground truth.

### 2.5 Expert verification

Two echocardiography experts, each with over 5 years of experience, analyzed the same images using an EchoPAC system (EchoPAC PC, version 203; GE Healthcare, Milwaukee Consway). They quantified the LVEDV, LVESV, and expert-EF using Simpson's biplane method.

To evaluate both intraobserver and interobserver variability, a subset of 25 patient datasets was randomly chosen and resubmitted to the experts for reanalysis using the same protocol. The experts recorded the analysis time and were blinded to the original EF results. Additionally, only one cardiac cycle was provided to ensure that all investigators analyzed the same heartbeat. The inter- and intra-observer variability were evaluated by intraclass correlation coefficients and the Bland–Altman test.

### 2.6 Statistical analysis

SPSS 25.0 software served as the primary tool for analysis in this study. Quantitative data underwent normality testing, and normally distributed data are presented as the mean ± standard deviation. The *t*-test was utilized for comparisons between groups. While the paired-samples sign test was performed for non-normally distributed data. Enumerated data are expressed as percentages. A significance level of *p* < 0.05 was considered statistically significant. The results for junior-EF and automated-EF were compared with expert-EF. The intra-group correlation coefficient (ICC) was calculated, and the Bland–Altman test was employed to assess consistency. Additionally, the Bland–Altman test was utilized to examine both inter- and intra-group agreement of the measurements conducted by clinical experts.

## 3 Results

Table 1 summarizes the characteristics of the study population in the training dataset. Table 2 presents the baseline clinical and echocardiographic data of the 29 patients. The diagnoses of this patient group were as follows: nine patients with fulminant myocarditis, twelve patients with severe pneumonia (including two cases of bronchopulmonary dysplasia, three cases of acute leukemia after chemotherapy, one case of cardiac tumors, and one case of thalassemia), three patients with myocardial injury in non-fulminant myocarditis (including one case each of splenic infarction, hepatoblastoma after chemotherapy, and hemorrhagic shock associated with intestinal infection), four patients with postoperative congenital heart disease (including one case each of single atrium, complete transposition of the great arteries, complete anomalous pulmonary venous connection, and aortic stenosis), and one patient with dilated cardiomyopathy.

**Table 1:** Characteristics of training data set.

n	80
Age, months	48.5 (22∼192)
Males,%	52 (65.0)
Height, cm	104 (58∼169)
Weight, kg	16.5 (5∼61)
Heart rate, bpm	98.5 (60∼128)
Ultrasound system,%	
Vivid E9	65 (81.75)
Vivid E95	15 (18.75)

**Table 2:** Clinical and echocardiographic data.

Patient demographics (*n* = 29)	
Age, months	57.5 (0∼160)
Males,%	13 (44.8)
Height, cm	106 (45∼160)
Weight, kg	16 (2.6∼53)
Heart rate, bpm	113.5 (12∼207)
Types of ECMO^a^,%	
Veno-Arterial	25 (86.2)
Veno-Venous	4 (13.8)
Prognosis,%	
Survival	24 (82.8)
Death	5 (17.2)
ECMO support time, day	
All	7.0（4.0∼15.5）
Survival	6.5（4.3∼15.5）
Death	7.0（1.5∼21.0）
Medical history,% (*n* = 29)	
Fulminant myocarditis	9 (31.0)
Severe pneumonia	12 (41.4)
Myocardial injury in non-fulminant myocarditis	3 (10.4)
Post-operation of congenital heart disease	4 (13.8)
Dilated cardiomyopathy	1 (3.4)
Ultrasound system,% (*n* = 218)	
GE Vivid E9	86 (39.5)
GE Vivid E95	132 (60.5)
Heart rate, bpm (*n* = 218)	
Pre -ECMO (*n* = 25)	134（73∼207）
ECMO (*n* = 128)	117（12∼168）
Post-ECMO (*n* = 65)	109（70∼171）

^a^ ECMO, extracorporeal membrane oxygenation.

### 3.1 Performance of PEQ-Net in LV segmentation on local data sets

**Figure 2:**
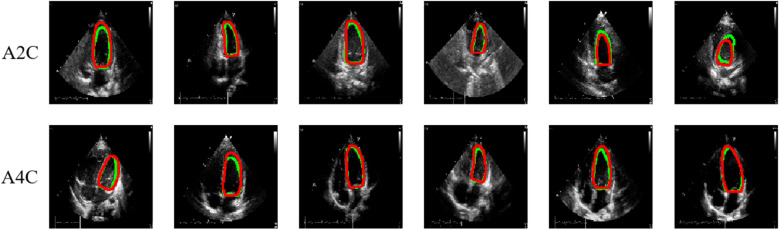
Quantitative results of PEQ-Net in LV segmentation. The red and green contour lines represent the ground truth and predicted masks in segmentation, respectively.

**Figure 3:**
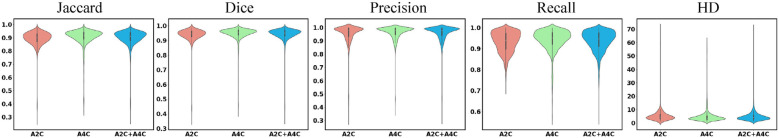
High performance of the PEQ-Net for segmentation in all frames. The *x*-axis represents the different views, i.e., A2C, A4C, and A2C + A4C. The *y*-axis represents each index. From left to right, each column represents the different indices, i.e., Jaccard, Dice, Precision, Recall and HD.

**Figure 4:**
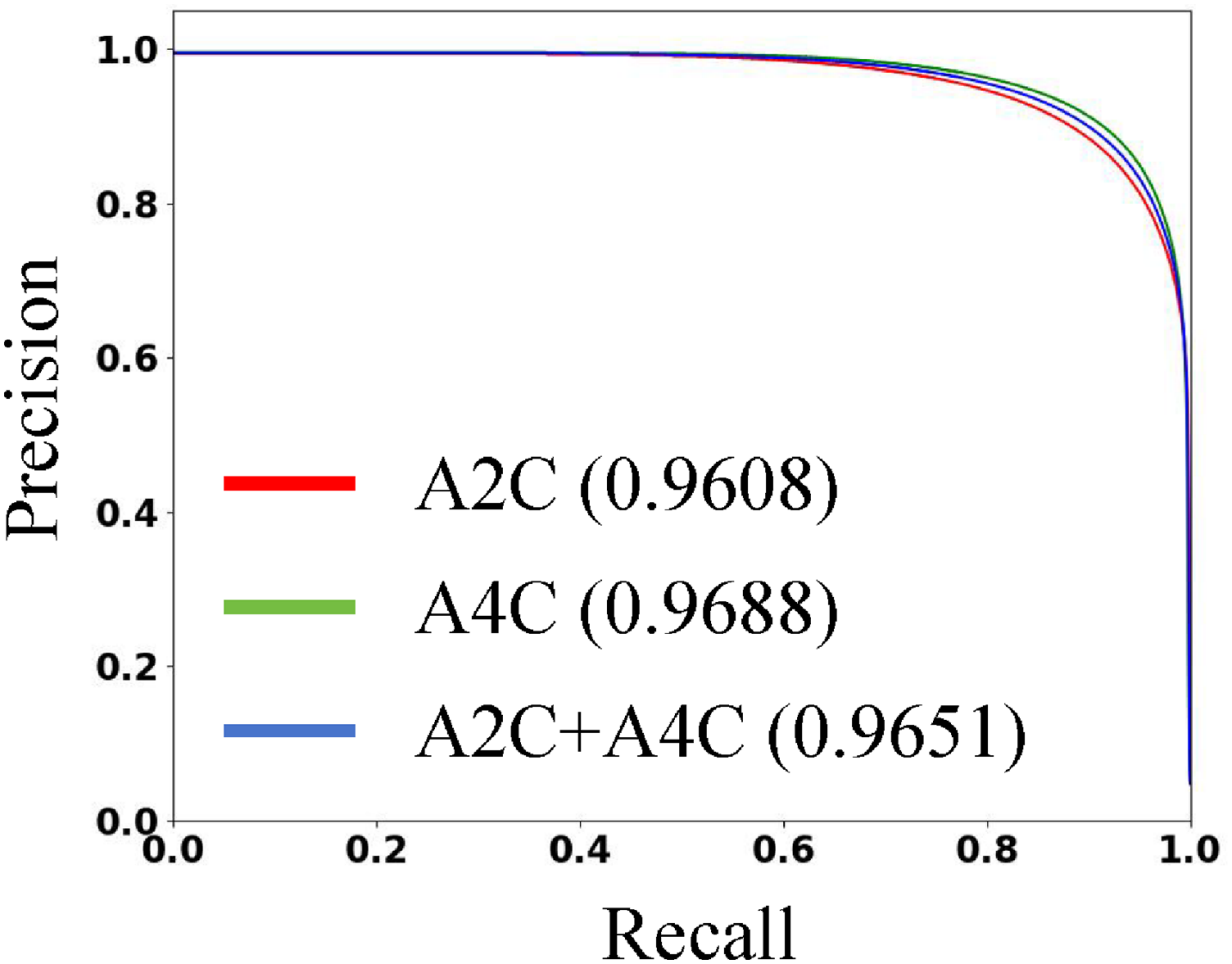
The *P*-R curve of PEQ-Net in LV segmentation. The red line represents the A2C view. The green line represents the A4C view. The blue line represents both A2C and A4C views. We use the area under the curve to evaluate the performance.

**Figure 5:**
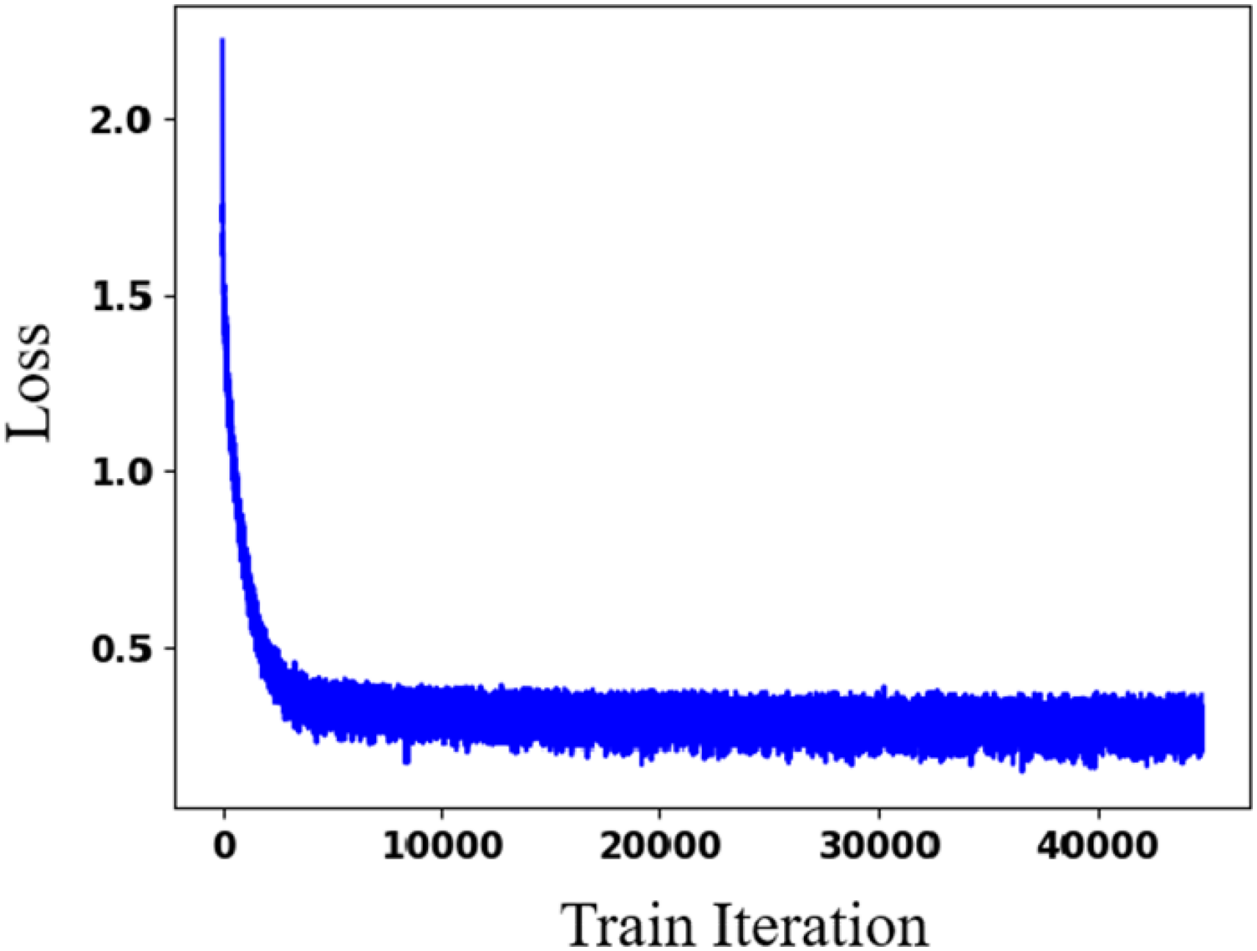
Effectiveness evaluation of PEQ-Net in LV segmentation. The *x*-axis is the training iteration. The *y*-axis is the loss function.

**Figure 6:**
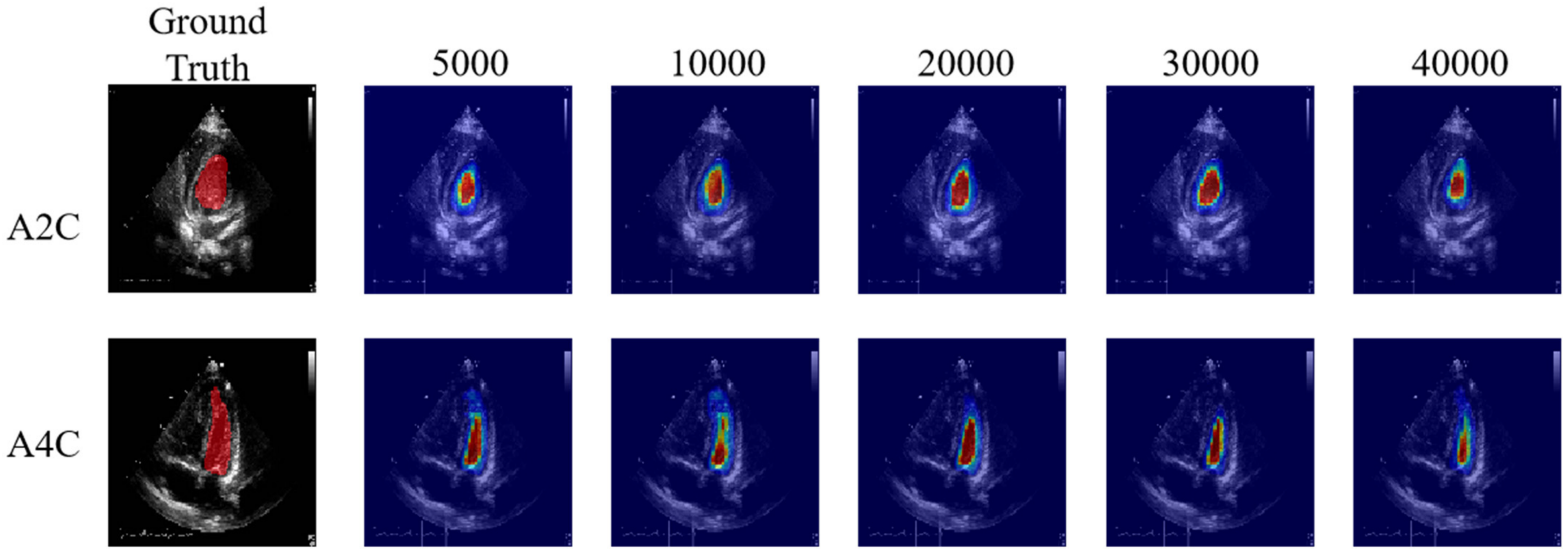
High agreement between the detected regions by the PEQ-Net and the region of interest. From left to right, each column indicates the attention map across different training iteration (5,000, 10,000, 20,000, 30,000, 40,000).

Table 3 presents the quantitative results of PEQ-Net in LV segmentation. The segmentation performance of PEQ-Net is assessed using metrics including Jaccard, Dice, and HD, with corresponding values of 0.8994, 0.9407, and 5.0885, respectively. Figure 2 visually illustrates the qualitative comparison of PEQ-Net in LV segmentation. This visual analysis affirms that PEQ-Net achieves precise segmentation of the LV region in ultrasound sequences. Moreover, Figures 3, 4 provide a comprehensive evaluation of PEQ-Net's performance across multiple metrics, including Jaccard, Dice, Precision, Recall, and HD. These figures underscore the high performance of PEQ-Net in LV segmentation. Figure 5 demonstrates how PEQ-Net effectively facilitates the convergence of the loss function, indicating stable and efficient training. Figure 6 illustrates the strong agreement between the regions detected by PEQ-Net and the region of interest, further validating the model's accuracy in LV segmentation.

**Table 3:** Performance of the PEQ-Net for segmentation in all frames.

	Jaccard	Dice	Precision	Recall	HD	Specificity	FPR	Accuracy
A2C	0.8903	0.9344	0.9531	0.9319	5.5760	0.9963	0.0037	0.9896
A4C	0.9081	0.9465	0.9623	0.9418	4.6312	0.9967	0.0033	0.9907
A2C + A4C	0.8994	0.9407	0.9579	0.9370	5.0885	0.9965	0.0035	0.9902

### 3.2 Assessment of different methods for determining Ef

Tables 4, 5 presents the LVEDV, LVESV and EF results of these three groups and their comparisons. The average time required for automated EF analysis and manual measurement was 16.11 ± 11.62 and 151.23 ± 3.65 s per case, respectively (analyzed in 25 views).

**Table 4:** LVEDV and LVESV assessed using various methods.

All(*n* = 186)	Junior	Automated	Expert	Z/*p*		
				Junior—Automated	Junior—Expert	Automated—Expert
LVEDV (ml)	33.77 (18.17∼57.57)	35.23 (19.50∼53.26)	34.90 (18.00∼53.11)	−4.001/0.000*	−8.625/0.000*	−0.479/0.632
LVESV (ml)	12.27 (6.46∼25.26)	14.73 (7.79∼26.73)	14.00 (7.00∼24.25)	−5.311/0.000*	−9.043/0.000*	−0.356/0.722

*The significance was set at a *p*-value <0.05 in the study.

**Table 5:** Efs assessed using various methods.

	Junior-EF	Automated-EF	Expert-EF	Z/*p*		
				Junior—Automated	Junior—Expert	Automated—Expert
All (*n* = 186)	60.0 (35.0∼65.0)	56.2 (31.9∼62.7)	58.0 (33.5∼62.0)	−5.598/0.000*	−4.794/0.000*	−1.767/0.079
EF ≤ 40 (*n* = 60)	28.0 (20.2∼35.0)	23.5 (20.2∼30.9)	24.5 (21.0∼31.8)	−3.740/0.000*	−3.038/0.002*	−1.737/0.082
EF > 40 (*n* = 126)	63.0 (60.0∼67.0)	59.2 (58.0∼62.3)	60.0 (58.0∼63.0)	−4.188/0.000*	−3.712/0.000*	−1.735/0.083
HR ≤ 140 (*n* = 154)	60.5 (35.0∼65.0)	56.6 (32.3∼63.3)	58.5 (33.0∼62.0)	−5.099/0.000*	−4.342/0.000*	−1.881/0.070
HR > 140 (*n* = 32)	59.5 (30.7∼64.7)	55.2 (31.2∼60.5)	55.5 (31.7∼59.7)	−2.300/0.021*	−2.021/0.043*	−1.552/0.121
PRE-ECMO (*n* = 20)	61.5 (47.5∼66.7)	56.6 (42.5∼62.4)	57.5 (43.5∼62.3)	−2.427/0.015*	−1.999/0.046*	−1.680/0.093
ECMO support (*n* = 109)	47.0 (26.0∼63.5)	39.8 (23.1∼59.1)	40.0 (23.0∼60.0)	−4.063/0.000*	−2.988/0.003*	−1.825/0.075
Post-ECMO (*n* = 57)	63.0 (61.0∼67.0)	59.1 (56.4∼62.8)	60.0 (58.5∼63.0)	−2.920/0.004*	−3.224/0.001*	−0.338/0.736

* The significance was set at a *p*-value < 0.05 in the study. EF, ejection fraction (%); HR, heart rate; ECMO, extracorporeal membrane oxygenation.

**Figure 7:**
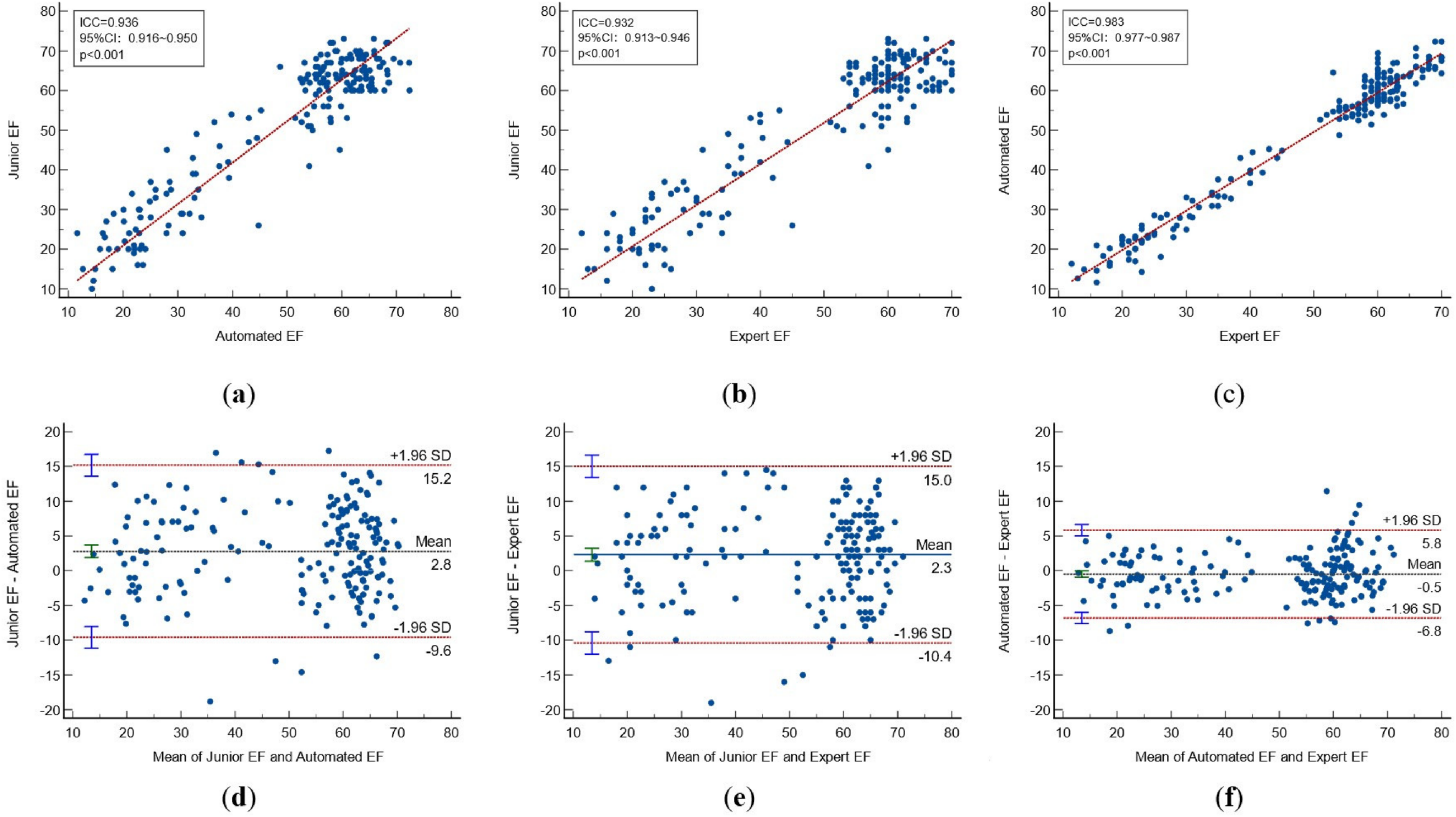
Correlations and bland-altman plots between different methods. (Top)Correlation plots and (Bottom)Bland-Altman plots between: automated EF and junior EF **(a,d)**; Expert and junior EF **(b,e)**; automated EF and expert EF **(c,f)**. CI, confidence interval; ICC, intraclass correlation coefficient; EF, sejection fraction.

Using the paired-samples sign test, there was significant difference between expert-EF and junior-EF in all groups. Junior-EF was consistently higher than expert-EF and automated-EF in all groups. However, no statistically significant differences were observed between automated-EF and expert-EF in all groups. Good correlations were observed between junior-EF, expert-EF, and automated-EF using ICCs (Table 6, Figure 7). Bland–Altman analysis revealed that the bias and limits of agreement were relatively lower between junior-EF and expert-EF (mean bias: 2.305%, 95% CI: −10.413 to 15.022%; Figure 7e) compared to those between expert-EF and automated-EF (mean bias: −0.490%, 95% CI: −6.812 to 5.831%; Figure 7f).

**Table 6:** Results of intra-group correlation and agreement analysis of EF in patients with ECMO.

	Junior EF (%)	Automated EF (%)	Expert EF (%)
Agreement: ICC (95% CI)			
Junior EF	1	–	–
Automated EF	0.936 (0.916∼0.950)*	1	–
Expert EF	0.932 (0.913∼0.946)*	0.983 (0.977∼0.987)*	1
Bland-Altman: bias (95% CI)			
Junior EF	0	–	–
Automated EF	2.795(−9.582∼15.172)	0	–
Expert EF	2.305(−10.413∼15.022)	−0.490 (−6.812∼5.831)	0

CI, confidence interval; EF, ejection fraction; ICC, intraclass correlation coefficient.

* *p* < 0.001.

### 3.3 Intra-observer and inter-observer variability

Out of 186 echocardiographic examinations, 25 patients were randomly chosen for the intra-observer and inter-observer repeatability test. The EF was measured repeatedly by clinical experts. Overall, there were no statistically significant differences between the intra-observer and inter-observer results in expert-EF (all *P* > 0.05), as shown in Table 7.

**Table 7:** Interobserver and intraobserver reliability of EFs measurements in expert and artificial intelligence.

(*n* = 25)	Expert EF^a^ (%)
Intraobserver Variability	
Reading 1	47.5 (28.0∼60.0)
Reading 2	48.5 (27.5∼59.7)
*p*	0.889
ICC	0.984 (0.970∼0.994)
Bland-Altman	0. 500(−5.013∼6.013)
Interobserver Variability	
Research 1	61.0 (60.0∼65.2)
Research 2	61.0 (60.0∼65.5)
*p*	0.701
ICC^a^	0.935 (0.842∼0.974)
Bland-Altman	0.083(−2.148∼2.315)

^a^ EF, ejection fraction; ICC, intraclass correlation coefficient.

## 4 Discussion

The primary finding of this study indicates that employing automated AI for capturing ED and ES images and measuring EF is technically feasible in pediatric patients. Our results demonstrated that PEQ-Net achieved high accuracy in LV segmentation, with high reproducibility and consistency indicated by the high Dice coefficients. The AI approach closely aligns with manual tracking performed by echocardiography experts, outperforming assessments made by junior physicians. Moreover, automated image analysis requires minimal time and provides informative results that exhibit a high correlation with expert standard measures, particularly in critically ill pediatric patients.

In pediatric clinical practice, pediatric cardiologists utilize echocardiography to assess cardiac structure, function, and hemodynamics. Functional echocardiography involves the bedside use of cardiac ultrasound to track functional and hemodynamic changes over time. Echocardiography plays a crucial role in the management of patients undergoing ECMO treatment, by providing information that helps physicians decide when to intubate, guide catheter placement, monitor progression, detect complications, and facilitate weaning of ECMO support (15). EF serves as a vital ECMO indication and prognostic echocardiographic marker, guiding treatment decisions and monitoring cardiac function in patients with heart failure during ECMO support. During ECMO weaning trials, the capability to perform real-time and expedited assessments of left ventricular function post-adjustment of the flow rate is crucial. This enables the attending physician to swiftly implement targeted interventions, thereby minimizing patient morbidity.

Bedside echocardiography provides clinicians with a wealth of hemodynamic information, offering insights into clinical conditions beyond assumed underlying physiology. Without bedside echocardiography, clinicians may only speculate about the underlying pathophysiology of compromised circulation (16), often leading to incorrect assumptions. To fully realize its clinical potential, bedside echocardiography must be readily available in the pediatric intensive care unit, allowing for immediate access. The reliability of pediatric echocardiography, particularly in critical care settings, is closely linked to the proficiency and expertise of practitioners. However, limited access to appropriate training programs and inter-disciplinary politics restrict the utilization of this potentially valuable clinical information. The dramatic increase in the use of echocardiography and the consequent increase in the use of echocardiography by pediatric intensive care unit physicians have exceeded the capacity of adequate training (17–21). When decisions are made based on EF, treatment of a subset of patients may be confounded due to the large variability in EF measurements between operators (22, 23). Especially when the heart rate of infants and young children is relatively fast, and when the bedside test of a critically ill patient requires rapid completion, the measured EF results may be highly variable. Consequently, there has been interest in AI-assisted automated measurement tools that can facilitate the assessment of LV function and minimize variability between echocardiographers.

Several automated AI methods have been employed in echocardiography studies for cardiac function testing (24–26). These studies used either semi-automatic EF measurement or required manual correction, resulting in variable results and increased measurement times (27). In contrast, our study achieved rapid EF determination in pediatric patients using echocardiography. The software algorithm utilized in this study eliminates the need for manual enveloping of the endocardial border. By simply inputting dynamic A4C and A2C views, the model automatically captures end-diastolic (ED) and end-systolic (ES) frames and calculates left ventricular end-diastolic volume (LVEDV), left ventricular end-systolic volume (LVESV), and ejection fraction (EF). This streamlined process takes only a few dozen seconds to complete the measurement, demonstrating superior temporal efficiency compared to previously described methods.

In this paper, we present PEQ-Net, a segmentation and quantification framework that allows multi-scale information extraction and deep supervision. PEQ-Net facilitates the unified modeling of multi-view echocardiographic sequences, effectively extracting and fusing multi-level and multi-scale holistic semantic features. It demonstrates outstanding generalization and robustness while remaining adaptable to heterogeneous data. Clinical evaluation experiments reveal that PEQ-Net achieves favorable results for both adult and pediatric datasets, displaying stability and coherence specifically in pediatric ECMO echocardiographic data. Compared with junior echocardiographers, it is closer to the performance of clinical experts.

In pediatric intensive care bedside echocardiography, inter-observer variability has always been a concern of the industry, especially because children on ECMO have different clinical states among critically patients in the ICU. The identification of the endocardium is more difficult, and it increases the intra-observer variability (28). The variability in EF evaluations of children in intensive care influences decision-making for ECMO and the management of ECMO weaning. This study leveraged PEQ-Net to obtain automatic AI-assisted EF measurements throughout the ECMO process in children, yielding results superior to those of junior physicians and closely resembling those of echocardiography experts. AI-assisted automated echocardiography facilitates the accuracy of bedside assessments and interpretation of cardiac function, and can improve the management of critically ill pediatric patients.

Performance benefit has different situations when the study adopts more advanced network architectures. First, adopting more advanced network architectures directly might bring declined performance. The architecture may not apply to pediatric echocardiography. Second, more advanced networks with elaborative modification might have better performance. Researchers design deep learning networks according to the challenges of datasets. Third, adopting more advanced network architectures might be a trade-off choice. More advanced network architectures might bring performance benefits in some aspects. The architecture might also lose some performance in other aspects.

## 5 Research limitations

This study has limitations. First, while previous research has indicated the superior estimation of left ventricular (LV) function using 3D echocardiography, our study did not assess 3D technology, which has the potential to provide more accurate evaluations (26). Second, the exclusion of patients with atrial fibrillation and arrhythmia from our study may limit the generalizability of our findings. Further investigations are necessary to assess the performance of the software in patients with arrhythmias and irregular heartbeats. Although we successfully utilized AI-assisted echocardiography to measure ejection fraction (EF) in children with severe diseases, particularly during ECMO weaning recovery, and we evaluated intra-observer variability, additional study subjects are required for further validation. Future studies should include a more diverse range of subjects, encompassing healthy children and those with different diseases. While the inclusion of this section is not mandatory, it can be added to the manuscript if the discussion extends into unusually long or complex territory.

## 6 Conclusions

AI-automated analysis of echocardiography for quantifying left ventricular function in critically ill children with ECMO is quicker than assessments by junior doctors. Machine learning-enabled pediatric echocardiography image analysis for automated assessment of ejection fraction is feasible and provides results comparable to a manual determination by an echocardiography expert.

## Data Availability

The raw data supporting the conclusions of this article will be made available by the authors, without undue reservation.
